# Unraveling the role of PPFIA1 in cancer: a comprehensive multi-omics analysis and functional validation from pan-cancer to pancreatic cancer

**DOI:** 10.3389/fonc.2026.1723550

**Published:** 2026-07-08

**Authors:** Shu Li, Hailin Jiang, Xiaojia Li, Tingting Jiang, Keping Xie

**Affiliations:** Center for Pancreatic Cancer Research, The South China University of Technology School of Medicine, Guangzhou, Guangdong, China

**Keywords:** biomarker, immunotherapy, multi-omics, pancreatic cancer, PPFIA1

## Abstract

**Background:**

PTPRF interacting protein alpha 1 (PPFIA1), a cytoplasmic scaffold protein of the liprin family, modulates cell adhesion, signal transduction, and cytoskeletal dynamics. Its pan-cancer prognostic implications and functional roles, particularly in pancreatic cancer, remain underexplored.

**Methods:**

Pan-cancer datasets including TCGA, GTEx and TISCH were integrated to assess PPFIA1 expression, genomic alterations, and pathway enrichment via Kaplan-Meier survival analysis, Cox regression, cBioPortal, and GSEA. In pancreatic cancer, external cohorts (*e.g.*, GSE28735, GSE52452) were used for expression and prognosis validation. Besides, immunotherapy response was analyzed by using TIGER cohorts. Functional impacts were determined by using ShRNA-mediated knockdown in CFPAC-1, PANC-1 and Panc-02 cells and evaluating their proliferation, migration and invasion *in vitro* and *in vivo*.

**Results:**

PPFIA1 exhibited differential expression across multiple cancer types, with overexpression in pancreatic adenocarcinoma (PAAD) versus non-tumor tissue at mRNA/protein levels, predominantly in malignant cells. High PPFIA1 correlated with adverse prognosis in PAAD across multiple endpoints. Genomic analyses revealed amplifications in head/neck cancers and mutations in endometrial carcinoma, clustering in SAM domains. GSEA enrichment analysis indicated pan-cancer activation of the mitotic spindle assembly pathway and Epithelial-Mesenchymal Transition (EMT) pathways in PAAD. Knockdown suppressed PAAD cell proliferation, colony formation, migration, invasion, and tumor growth *in vivo*. Notably, elevated PPFIA1 predicted superior responses to immunotherapy in pan-cancer cohorts rather than pancreatic cancer.

**Conclusions:**

PPFIA1 emerges as a pan-cancer biomarker with prognostic significance and shows an oncogenic role in PAAD, potentially associating with EMT-related malignant phenotypes. Its association with immunotherapy efficacy suggests PPFIA1 warrants further investigation as a candidate biomarker and potential functional target for precision oncology.

## Introduction

Pancreatic cancer is a malignant tumor characterized by poor prognosis and high mortality. In recent years, its incidence has been increasing annually (1, 2), and it is projected to become the second leading cause of cancer-related deaths by 2030. The median survival time for pancreatic cancer is approximately 4 months, with a 5-year survival rate of 13% (3, 4). The primary reason for the low survival rate is the absence of early symptoms, resulting in most cases being diagnosed at an advanced stage (5). Currently, the most effective treatment is surgical resection of the tumor area; however, less than 20% of patients are eligible for surgery (6, 7). Most advanced pancreatic cancers are highly invasive or have already metastasized, resulting in the loss of the optimal opportunity for surgical treatment (8). For patients with inoperable pancreatic cancer, neoadjuvant therapy may convert the tumor to a resectable state. Although novel drug combinations and multimodal treatment regimens have significantly prolonged the survival of pancreatic cancer patients, approximately 80% of patients experience recurrence after surgery and eventually die from pancreatic cancer (9). Therefore, identifying novel biomarkers or therapeutic targets for pancreatic cancer could provide new directions for early diagnosis and effective treatment.

PTPRF interacting protein alpha 1 (PPFIA1), also known as liprin-α1, is a member of the liprin family of cytoplasmic scaffold proteins that interact with LAR protein tyrosine phosphatases. Liprins are cytoplasmic scaffold dimers classified into two subtypes, liprin-α and liprin-β, based on sequence similarity and predicted structural homology (10, 11). Vertebrates possess six liprin genes encoding four liprin-α (liprin-α1–α4) and two liprin-β (β1 and β2) proteins (11). PPFIA1 co-localizes with LAR at focal adhesions and mediates cell–extracellular matrix interactions by regulating focal adhesion disassembly (12). PPFIA1 plays a role in controlling synapse formation and function in neuronal cells, such as modulating synaptic maturation, synaptic vesicle trafficking, and neurotransmitter release (13–16). Studies have shown that PPFIA1 is overexpressed in various malignancies, including breast, ovarian, colon, and oral cancers (16–20). Aberrant expression of PPFIA1 plays a crucial role in tumor invasion and metastasis (21–23). These findings suggest that PPFIA1 may be a potential target influencing multiple cancer types. However, systematic evaluations of the prognostic and therapeutic roles of PPFIA1 in pan-cancer remain lacking.

This study utilized public databases to analyze the differential expression of PPFIA1 in various cancers and normal tissues. We evaluated the prognostic value, genomic alterations, and pathway enrichment of PPFIA1 across pan-cancers. Focusing on pancreatic cancer, we demonstrated that PPFIA1 serves as a potential molecular marker by examining its expression and prognostic significance. The impact of PPFIA1 on malignant phenotypes in pancreatic cancer was further validated through *in vivo* and *in vitro* experiments. Additionally, PPFIA1 was found to be associated with immunotherapy response in a pan-cancer manner. Our systematic analysis underscores the importance of PPFIA1 in cancer management.

## Materials and methods

### Collection and processing of pan-cancer data

The UCSC Xena platform served as the primary resource for retrieving data from the TCGA and GTEx databases, with a focus on evaluating PPFIA1 expression profiles. Single-cell RNA sequencing (scRNA-seq) data spanning various cancers were acquired from the TISCH database (24). For protein-level analysis, pan-cancer proteomic datasets were sourced from the Clinical Proteomic Tumor Analysis Consortium (CPTAC) and the International Cancer Proteome Consortium (ICPC). Spatial transcriptomic (ST) datasets were obtained from the original study by Moncada et al. (GSE111672). These multi-omics data were systematically integrated and visualized, with CPTAC, ICPC, and GSE111672 as original data sources (Sparkle database, https://grswsci.top/). External datasets specific to PAAD were sourced from GSE28735, GSE52452, GSE71729, GSE21501, ICGC PAAD AU, ICGC PAAD CA, and E-MTAB-6134. Probe mapping was performed using the “AnnoProbe” R package, and where multiple probes were present, average values were computed employing the “limma” R package as needed. To mitigate batch effects, data normalization was performed utilizing the ComBat algorithm implemented within the sva R package, ensuring robust correction of technical variations across datasets (25).

### Prognostic evaluation

The optimal expression threshold for PPFIA1 was established using the survminer R package, enabling the classification of samples within each cohort into elevated- and reduced-expression subgroups. This cutoff strategy is widely used to maximize discriminative performance in transcriptome-based survival analysis.The prognostic relevance of PPFIA1 in pan-cancer patients was assessed through Kaplan-Meier (KM) survival analysis, alongside univariate Cox proportional hazards models, employing the survival R package. The proportional hazards assumption was visually inspected and satisfied for all models. Additionally, external data validation primarily relies on the HPA and BEST web tools (https://journalofbigdata.springeropen.com/articles/10.1186/s40537-023-00844-y).

### Genomic landscape

Comprehensive assessments of genomic mutation frequencies, amplifications, and deep deletions across pan-cancer cohorts were conducted using the Cancer Type Summary module within the cBioPortal platform (26). Processed single nucleotide variation (SNV) data were subjected to detailed analysis with the “maftools” R package to characterize the mutational profile of PPFIA1 across diverse cancer types (27). Additionally, copy number variation (CNV) data, sourced from the GSCA database, were visualized and interpreted using the “ggplot2” R package.

### Functional enrichment analysis

In the TCGA pan-cancer cohort, samples were stratified into high-expression (top 30%) and low-expression (bottom 30%) groups based on gene expression levels. This threshold was chosen to balance group size, statistical power, and robustness against outliers. Differential expression analysis was conducted using the limma R package to calculate log2 fold change (log2FC) for each gene, followed by ranking genes according to their log2FC values. Gene set enrichment analysis (GSEA) was performed using the clusterProfiler R package (28) and visualized with the GseaVis R package (http://dx.doi.org/https://doi.org/10.1002/mdr2.70000), leveraging the hallmark gene set to identify and illustrate enriched biological pathways.

### Evaluation of immunotherapy response

Datasets pertaining to immunotherapy were sourced from the Tumor Immunotherapy Gene Expression Resource (TIGER). These cohorts (including IMvigor210 (urothelial carcinoma), VanAllen (melanoma), and Kim (melanoma)) represent multiple cancer types (e.g., urothelial carcinoma and melanoma) rather than pancreatic cancer–specific cohorts. Transcriptomic profiles were analyzed to explore the association between PPFIA1 expression and immunotherapy response across cancers. Correlations between PPFIA1 and tumor microenvironment indicators including immune cell infiltration, PD-L1, TMB, and MSI were not analyzed in this study. Pancreatic cancer is not an immunotherapy-responsive cancer, and the relevance of these findings to pancreatic cancer is limited and exploratory.

### Cell Lines and cell culture

The human pancreatic cancer cell lines CFPAC-1 and PANC-1 were purchased from Guangzhou Cellcook Biotech Co., Ltd (Guangzhou, China). The mouse pancreatic cancer cell line Panc02 was obtained from Meisen Cell Technology Co., Ltd (Zhejiang, China). CFPAC-1 cells were cultured in IMDM medium (Gibco), while PANC-1 and HEK-293T cells were maintained in DMEM medium (Gibco). Panc02 cells were grown in RPMI-1640 medium (Gibco). All media were supplemented with 10% fetal bovine serum (FBS) and 1% penicillin/streptomycin. Cells were incubated at 37 °C in a humidified atmosphere containing 5% CO₂. All cell lines were acquired between 2020 and 2022 and were routinely tested for mycoplasma contamination via PCR within the past six months.

### Plasmids and transfection reagents

The pLKO.1-U6-EF1a-copGFP-T2A-puro (Sh-NC), pLKO.1-U6-Sh-human-PPFIA1-EF1a-copGFP-T2A-puro (Sh-human-PPFIA1; ShRNA sequence: GGUCGACAGUUUUCACAGA), and pLKO.1-U6-Sh-mouse-PPFIA1-EF1a-copGFP-T2A-puro (Sh-mouse-PPFIA1; ShRNA sequence: AUGAUGUGCGAGGUGAUGC) plasmids were purchased from Ige BIOTECHNOLOGY Co., Ltd (Guangzhou, China). The target sequences for human and mouse PPFIA1 were selected based on previously validated efficacy in the literature to ensure reliable knockdown. All transfections were performed using Lipofectamine 3000 (Cat# L3000015, Invitrogen).

The Sh-PPFIA1 or Sh-NC lentiviral vectors were co-transfected with the psPAX2 and pMD2.G packaging plasmids into HEK-293T cells using Lipofectamine 3000. Lentiviral particles were harvested 48 hours after transfection. CFPAC-1, PANC-1, and Panc02 cells were infected with the optimal viral titer in medium containing 8 μg/mL polybrene (Cat# C0351, Beyotime). After 72 h of infection, stable polyclonal cell populations were selected with 2–4 μg/mL puromycin (Cat# ant-pr-1, InvivoGen) for one week and subsequently expanded.

### RNA extraction and quantitative real-time PCR

Total RNA was extracted from cells using TRIzol™ Reagent (Cat# 9109, Takara). The concentration and purity of the isolated RNA were measured using a NanoDrop™ 2000 spectrophotometer. Subsequently, RNA was reverse-transcribed into cDNA using a PrimeScript™ RT Reagent Kit (Cat# RR047A, Takara). Real-time PCR was performed using the synthesized cDNA as the template. The relative gene expression levels were calculated using the 2^−ΔΔT^ method and normalized to the expression of the housekeeping gene GAPDH. The primer sequences used were as follows: human-PPFIA1, forward 5’-CTTAACCCAGGGGAAGTTACAC-3’ and reverse 5’-ATCCTAAGAGACCGCTCATGC-3’; human-GAPDH, forward 5’-GCGAGATCCCTCCAAAATCA-3’ and reverse 5’-ACTTCTCATGGTTCACACCC-3’; mouse-PPFIA1, forward 5’-GCCCAACAAGCCAGCGTCTTGGCAA-3’ and reverse 5’-GCGAGGAAGCAGGGTAGGGGGG-3’; and mouse-GAPDH, forward 5’-AAGGTGGTGAAGCAGGCATCTGAG-3’ and reverse 5’-GGAAGAGTGGGAGTTGCTGTTGAAGTC-3’. Protein-level validation of knockdown efficiency by western blot was not performed, and only one shRNA sequence was used for human and mouse PPFIA1.

### Cell viability assay

CFPAC-1, PANC-1, and Panc02 cells were seeded in 96-well plates at a density of 2–3x10^3^ cells per well. At the indicated time points, CCK-8 reagent (CAT# CK04, DOJIND) was added to each well, followed by incubation at 37 °C for 1–2 h. The absorbance at 450 nm was then measured using a microplate reader (Tecan, Zurich, Switzerland).

### Colony formation assay

CFPAC-1 (1,000 cells), PANC-1 (500 cells), and Panc02 (1,000 cells) were seeded into 6-well plates and cultured in complete medium for 10–14 days. The cell clones were fixed with paraformaldehyde, washed with PBS, and stained with crystal violet (CAT# C0121, Beyotime).

### Cell migration and invasion assay

Cell migration and invasion assays were performed using Transwell chambers (CAT# 3422, Corning). Briefly, 2–5x10^4^ cells were seeded into the upper chamber of the Transwell. The upper chamber contained serum-free medium, while the lower chamber was filled with medium supplemented with 10% serum. After 48 h of incubation, cells were stained with crystal violet for 15 min. The number of cells was quantified under a microscope.

### Syngeneic tumor growth model

All experiments involving mouse models were conducted in accordance with the Animal Experiment Guidelines of South China University of Technology. 8-week-old male C57BL/6 mice were purchased from Guangdong Sijiajingda Biotechnology Co., Ltd. All experimental animals were housed in the animal facility of South China University of Technology, which is accredited by the Association for Assessment and Accreditation of Laboratory Animal Care, and maintained in compliance with current regulations and standards.

A mixture of Panc02 cells (5x10⁵ per mouse) and BD Matrigel (1:1) was subcutaneously injected into the dorsal thigh of 8-week-old male C57BL/6 mice (n=5). The sample size of five mice per group was determined based on established protocols for subcutaneous xenograft models in pancreatic cancer research and in adherence to the “3Rs” principles (Replacement, Reduction, and Refinement) of animal ethics to minimize animal usage while maintaining sufficient power to detect consistent biological trends. Tumor length and width were measured every three days using a vernier caliper. Tumor-bearing mice were sacrificed by cervical dislocation on day 24 after inoculation, and tumors were excised for further analysis. Tumor volume (mm^3^) was calculated using the formula: 0.5 x length x width².

### Statistical analysis

Data analysis and visualization were conducted using R version 4.3.1, with additional visualizations performed via the Sanger Box bioinformatics analysis online tool. Statistical analyses were executed using GraphPad Prism 8.0 software under a licensed agreement. Comparisons between two groups were made using the Wilcoxon rank-sum test. All statistical tests were two-sided, and p-values < 0.05 were considered statistically significant, with statistical significance indicated by *p < 0.05, **p < 0.01, ***p < 0.001 and ****p< 0.0001. Multiple-testing correction was applied where appropriate.

## Results

### Overview of PPFIA1 in pan-cancer multi-omics

**Figure 1:**
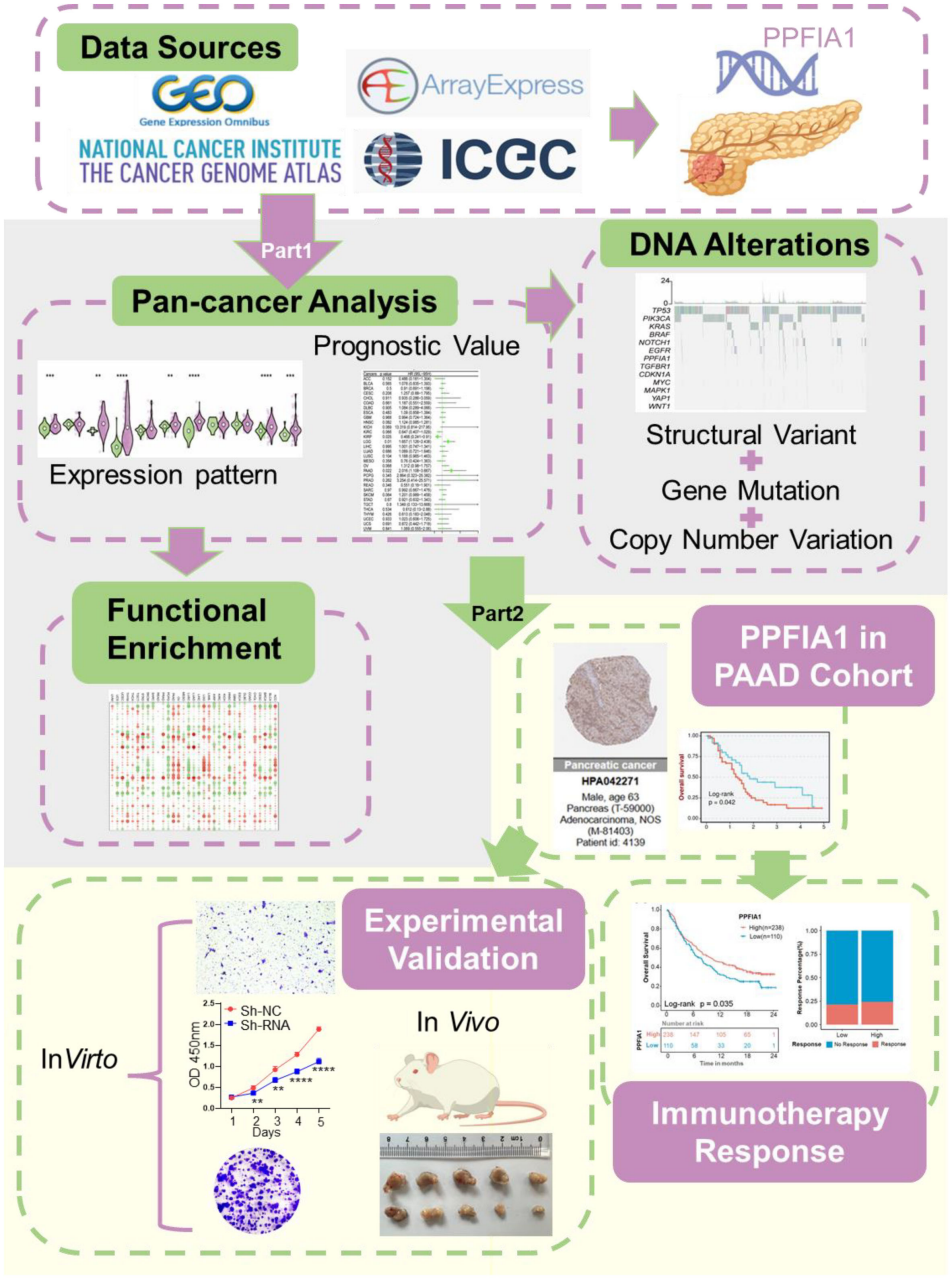
Flowchart. The present research included the uses of various public datasets and platforms for analyses of gene expression patterns and their relationship with patient prognosis, and uses of *in vitro* and *in vivo* experimental model for functional validation.

**Figure 2:**
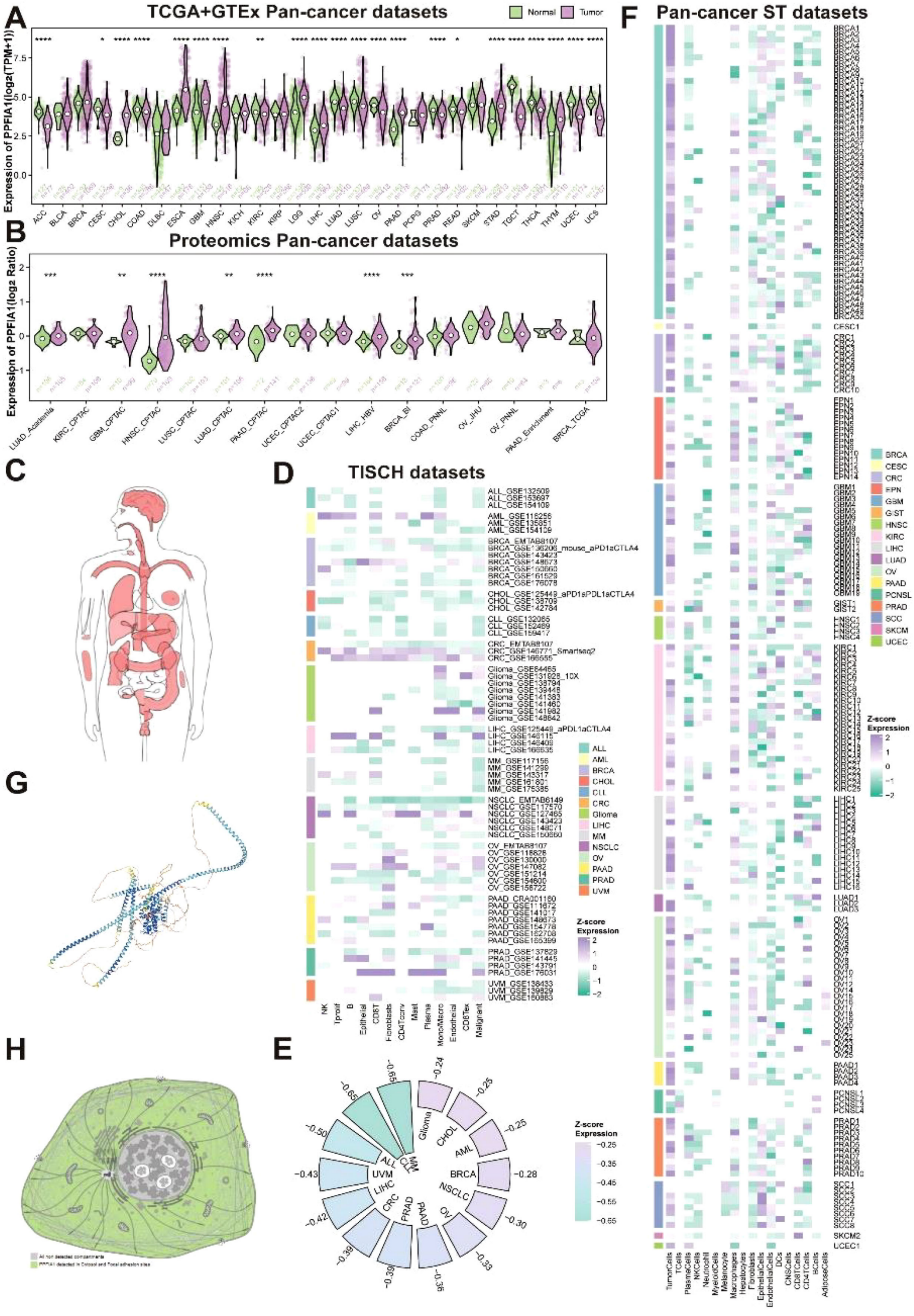
Overview of PPFIA1 expression patterns across pan-cancer multi-omics datasets. **(A)** Boxplot representation of PPFIA1 mRNA expression levels in pan-cancer datasets from TCGA and GTEx. **(B)** Boxplot of PPFIA1 protein expression ratios in pan-cancer proteomic datasets. **(C)** Schematic illustration of PPFIA1 mRNA expression mapped onto a human organ diagram. **(D)** Heatmap of PPFIA1 expression in single-cell transcriptomic data (TISCH dataset). **(E)** Pie chart showing the relative expression of PPFIA1 across different cancer types. **(F)** Heatmap of PPFIA1 expression in pan-cancer spatial transcriptomic (ST) datasets. **(G)** Structural representation of the PPFIA1 protein conformation. **(H)** Schematic of a cell showing the primary subcellular localization of PPFIA1. *p < 0.05, **p < 0.01, ***p < 0.001, **p < 0.0001.

Our research flowchart is illustrated in **Figure 1**. We initially investigated the expression profile of PPFIA1 at the mRNA level across pan-cancer datasets. As shown in **Figure 2A**, by integrating data from TCGA and GTEx projects, we observed differential expression of PPFIA1 between tumor and normal tissues in most cancer types, with the exception of BLCA, BRCA, DLBC, KICH, KIRP, PCPG, and SKCM. Notably, PPFIA1 exhibited significantly higher expression in normal tissues for CESC, COAD, KIRC, LUAD, LUSC, OV, PRAD, READ, TGCT, THCA, UCEC, and UCS. Conversely, in CHOL, ESCA, GBM, HNSC, LGG, LIHC, PAAD, STAD and THYM, PPFIA1 was markedly overexpressed in tumor tissues. In proteomic analyses (**Figure 2B**), LUAD displayed an expression trend opposite to that observed at the mRNA level. In contrast, GBM, HNSC, PAAD, and LIHC exhibited consistent trends at both mRNA and protein levels, with elevated expression in tumor tissues. Mapping PPFIA1 expression from TCGA pan-cancer data onto a schematic of human organs revealed relatively uniform expression levels across different tissues (**Figure 2C**).

At the single-cell transcriptomic level, PPFIA1 expression was detected across various cell types within the tumor microenvironment. Notably, PPFIA1 was consistently expressed in cancer cells across multiple cancer types (**Figure 2D**), with particularly high expression in multiple myeloma (MM) and chronic lymphocytic leukemia (CLL) (**Figure 2E**). Spatial transcriptomic data further indicated that PPFIA1 expression is predominantly localized to cancer cells across pan-cancer samples, with negligible expression in myeloid cells (**Figure 2F**). Additionally, we characterized the protein conformation of PPFIA1 (**Figure 2G**), with its primary subcellular localization identified in the cytoplasm (**Figure 2H**).

**Figure 3:**
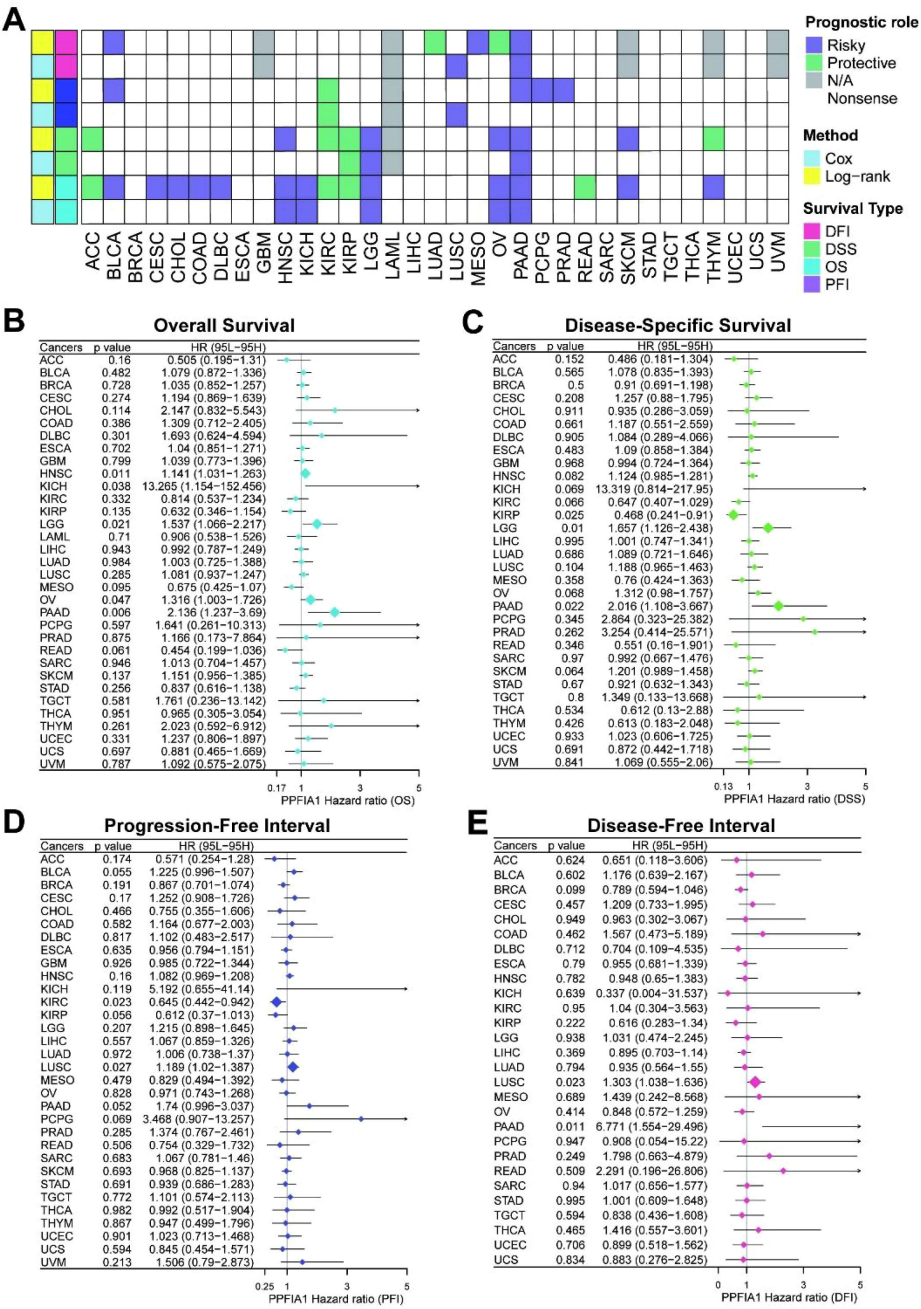
Prognostic significance of PPFIA1 across pan-cancer datasets. **(A)** Heatmap summarizing the prognostic role of PPFIA1 across pan-cancer datasets, based on Kaplan-Meier and Cox regression analyses. Colors indicate the prognostic risk (blue indicates high risk, green indicates protective effect, gray indicates non-significant, with methods (Cox or log-rank) and survival types (OS: overall survival, DSS: disease-specific survival, DFI: disease-free interval, PFI: progression-free interval) annotated. **(B–E)** Forest plot showing hazard ratios (HR) from univariate Cox regression analysis for OS/DSS/PFI/DFI.

Subsequently, we evaluated the prognostic implications of PPFIA1 across pan-cancer datasets, employing KM survival analysis and Cox regression methods, with the results summarized in **Figure 3A**. Based on univariate Cox regression analysis, with overall survival (OS) as the endpoint, KICH exhibited the highest hazard ratio (HR) with statistical significance (**Figure 3B**). In terms of disease-specific survival (DSS), PAAD displayed the highest statistically significant HR, while KIRP showed the lowest HR (**Figure 3C**). For progression-free interval (PFI), only KIRC demonstrated statistical significance, acting as a protective factor (**Figure 3D**). In the context of disease-free survival (DFS), PAAD was the only cancer type with statistical significance, identified as a risk factor (**Figure 3E**). Overall, as depicted in **Figure 3A**, the prognostic significance of PPFIA1 varies across different tumor types. Notably, PAAD consistently showed prognostic relevance across multiple endpoints, serving as a risk factor, although the P-value for PFI was of borderline significance (P = 0.052). In conclusion, the expression patterns of PPFIA1 underscore its potential as a biomarker, with particularly pronounced significance in PAAD. Collectively, these expression patterns suggest that PPFIA1 holds potential as a biomarker in cancers.

### Genomic alterations of PPFIA1 in pan-cancer

**Figure 4:**
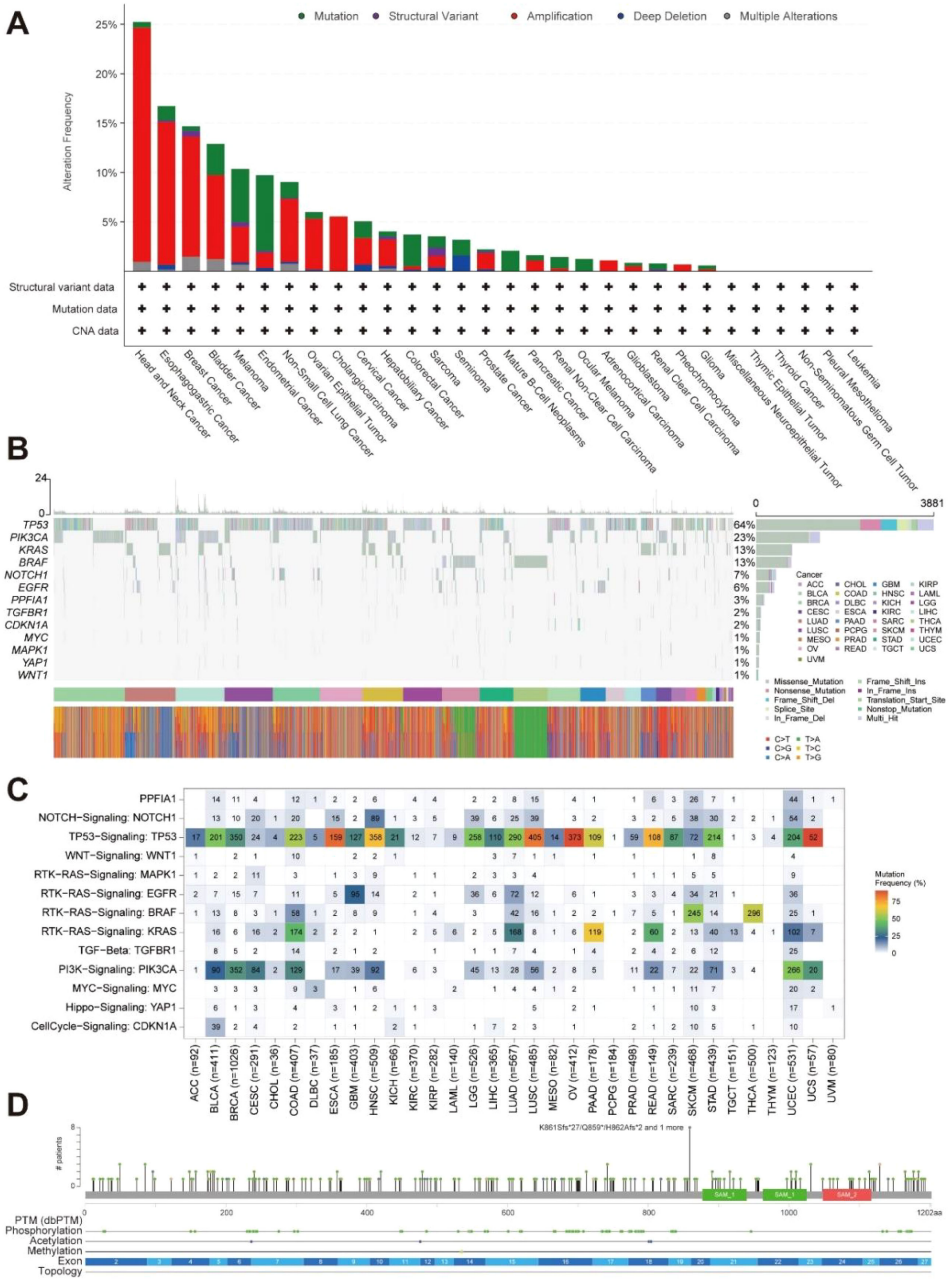
Genomic alterations of PPFIA1 across pan-cancer cohorts. **(A)** Stacked bar chart illustrating the frequency of different types of PPFIA1 genetic alterations across pan-cancer cohorts. **(B)** The waterfall plot illustrates the mutation frequencies of PPFIA1 alongside frequently mutated genes. **(C)** Heatmap displaying co-mutation frequencies between PPFIA1 and key genes (*e.g.*, TP53, KRAS) in oncogenic signaling pathways across pan-cancer types. **(D)** Detailed lollipop plot emphasizing mutation clusters on functional domains of the PPFIA1 protein, notably within sterile alpha motif (SAM) regions (approximately amino acids 800-1200).

Genomic modifications constitute a critical component in the development of tumor heterogeneity. Single nucleotide variations (SNVs), characterized by the substitution of a single nucleotide within the DNA sequence, are pivotal in facilitating tumor advancement. Similarly, aberrant copy number variations (CNVs) are increasingly recognized as essential molecular drivers underlying oncogenesis. Analysis of PPFIA1 genomic alterations revealed prominent amplification in head and neck cancer, followed by esophageal cancer, breast cancer, and bladder cancer (**Figures 4A–C**). In terms of mutations, only endometrial cancer exhibited a frequency exceeding 5%. Other alteration types, including structural variants, deep deletions, and multiple alterations, were observed at notably lower frequencies. The provided lollipop plot illustrates the mutation landscape of the PPFIA1 protein across various cancer types, spanning its 1202 amino acid (aa) sequence (**Figure 4D**). The x-axis represents the amino acid positions, while the y-axis indicates the number of patients exhibiting mutations at specific sites, with a maximum of 8 patients noted. The plot highlights the distribution of mutations, with notable clusters occurring within the sterile alpha motif (SAM) domains, labeled as SAM_1 and SAM_2, located approximately between 800–1000 aa and 1000–1200 aa, respectively. Specific mutations, such as K861Sfs*27/Q859*/H862Afs*2, are annotated, suggesting frameshift or truncating alterations.

### PPFIA1-mediated downstream pathways regulates tumor biology

Following the elucidation of PPFIA1’s diverse roles across various tumors, we proceeded to investigate the downstream signaling pathways through which it exerts its effects. To this end, we applied hallmark gene sets in a pan-cancer context and conducted GSEA. The results indicated that PPFIA1 predominantly activates the Mitotic Spindle pathway across most cancer types, followed by the UV Response Down pathway. Conversely, among the inhibited pathways, Oxidative Phosphorylation exhibited a consistent suppression across different malignancies. Pathways such as TNFA Signaling via NF-κB, Myogenesis, Interferon Gamma Response, and Interferon Alpha Response displayed variable activation patterns across cancer types. Notably, in pancreatic PAAD, our primary focus, the activation of the Epithelial-Mesenchymal Transition (EMT) pathway was most pronounced. The roles of mitotic spindle and oxidative phosphorylation pathways in pancreatic cancer were not further explored in this study.

### External datasets validates PPFIA1 as a novel biomarker for PAAD

**Figure 5:**
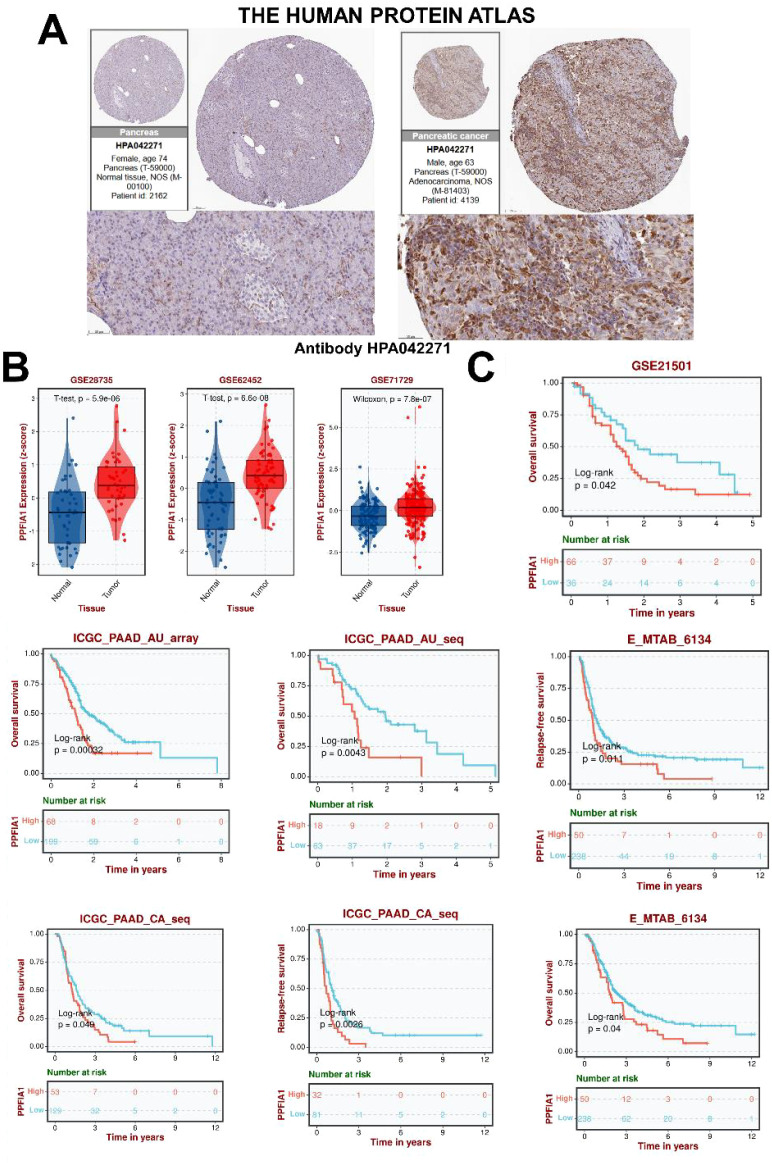
Validation of PPFIA1 as a potential biomarker in PAAD using external datasets. **(A)** Immunohistochemical staining images from The Human Protein Atlas (HPA) database, showcasing PPFIA1 protein localization in normal pancreatic tissue and PAAD tissue. **(B)** Boxplots showing PPFIA1 mRNA expression (Z-score) across independent external datasets. **(C)** Kaplan-Meier survival curves assessing the prognostic impact of PPFIA1 expression in external cohorts.

As depicted in **Figure 3A**, PPFIA1 emerges as a risk factor for prognosis across multiple endpoints in the TCGA-PAAD. To further assess its potential as a biomarker in PAAD, we conducted validation using several external datasets. Analysis of the HPA database revealed that PPFIA1 protein expression is predominantly localized to the cytoplasm, with significantly elevated levels in PAAD compared to benign pancreatic tissue (**Figure 5A**). Similarly, at the mRNA level, external datasets GSE28735, GSE52452, and GSE71729 consistently demonstrated higher PPFIA1 expression in PAAD (**Figure 5B**). Subsequently, we evaluated its prognostic relevance in additional external cohorts, including GSE21501, ICGC PAAD AU, ICGC PAAD CA, and E-MTAB-6134. As shown in **Figure 5C**, patients with elevated PPFIA1 expression exhibited poorer outcomes when OS or relapse-free survival (RFS) served as prognostic endpoints. These findings collectively suggest that PPFIA1 holds considerable promise as a novel biomarker in PAAD.

### PPFIA1 promotes the malignant phenotype of pancreatic cancer

**Figure 6:**
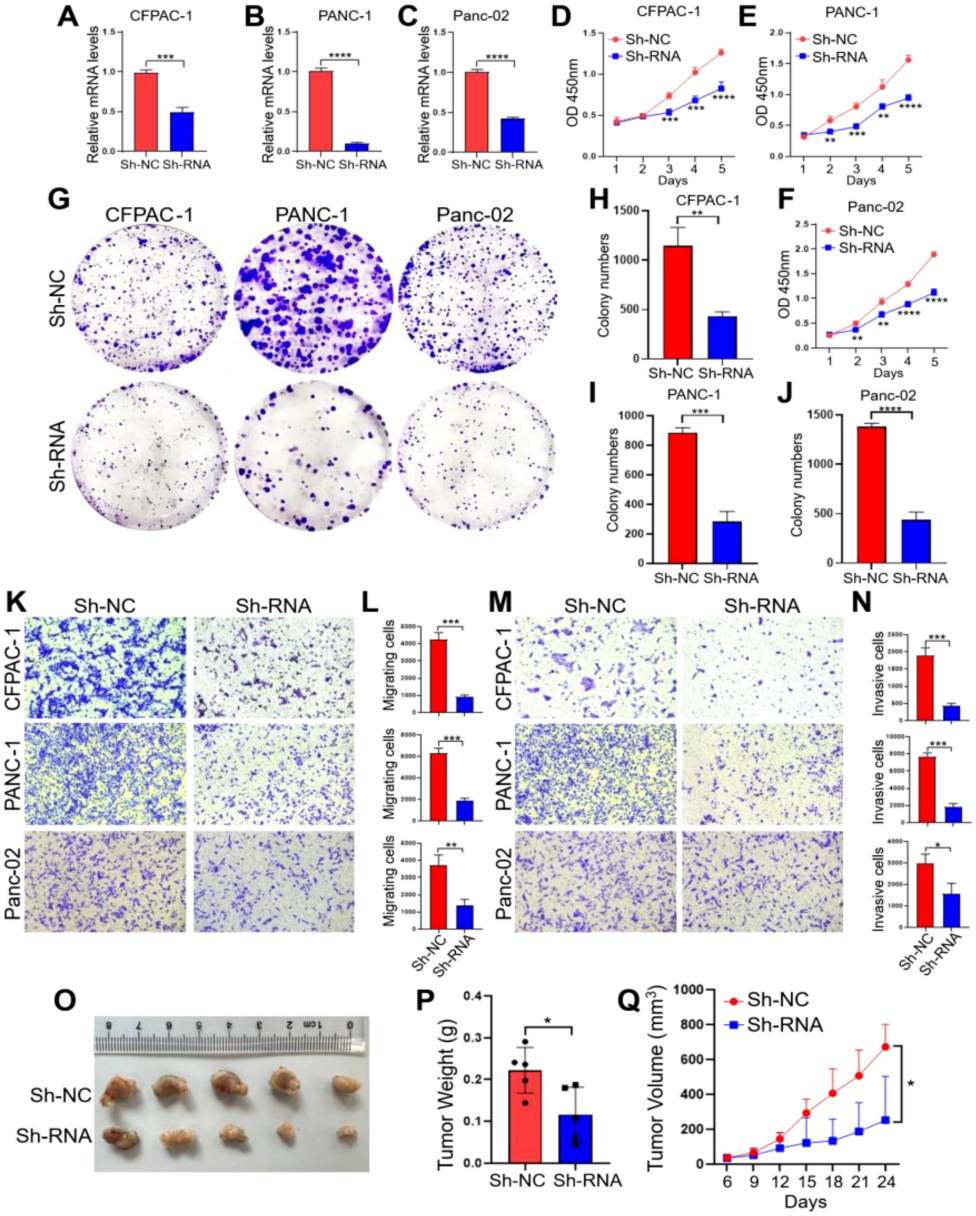
PPFIA1 promotes pancreatic cancer cell malignancy. **(A–C)** The mRNA expression level of PPFIA1 in CFPAC-1 **(A)**, PANC-1 **(B)**, and Panc02 **(C)** cells was analyzed by qPCR. **(D–F)** The effect of PPFIA1 knockdown on the cell viability of CFPAC-1 (**(D)**, PANC-1 **(E)**, and Panc02 **(F)** was analyzed by CCK-8 assay. **(G)** The effect of PPFIA1 knockdown on the proliferation of CFPAC-1, PANC-1, and Panc02 cells was analyzed by colony formation assay. **(H–J)** The quantitative analysis of the number of colonies in **(G)** was performed. **(K)** The effect of PPFIA1 knockdown on the migration of CFPAC-1, PANC-1, and Panc02 cells was analyzed using Transwell assay. **(L)** The quantitative analysis of cell numbers in **(K)** was conducted. **(M)** The effect of PPFIA1 knockdown on the invasion of CFPAC-1, PANC-1, and Panc02 cells was analyzed by Transwell assay. **(N)** The quantitative analysis of cell numbers in **(M)** was conducted. **(O–Q)** The effect of PPFIA1 knockdown on tumorigenicity was evaluated using Panc02 cells. Tumor photographs (n = 5) **(O)**, tumor weights **(P)**, and tumor volumes **(Q)**. *p < 0.05, **p < 0.01, ***p < 0.001, ****p < 0.0001.

To investigate the functional role of PPFIA1 in pancreatic cancer, we knocked down PPFIA1 in human (CFPAC-1 and PANC-1) and mouse (Panc02) pancreatic cancer cell lines. The efficiency of ShRNA-mediated knockdown was confirmed by measuring PPFIA1 mRNA levels using qPCR (**Figures 6A–C**). Subsequently, the effects of PPFIA1 on pancreatic cancer proliferation were evaluated using CCK-8 (**Figures 6D–F**) and colony formation assays (**Figures 6G–J**). The results showed that PPFIA1 knockdown significantly reduced cell viability and the number of single-cell colonies. Moreover, migration (**Figures 6K, L**) and invasion (**Figures 6M, N**) abilities were markedly impaired in CFPAC-1, PANC-1, and Panc02 cells upon PPFIA1 depletion. Consistent with the *in vitro* findings, a tumor formation assay using Panc02 cells with PPFIA1 knockdown further confirmed that PPFIA1 promotes pancreatic cancer proliferation (**Figures 6O–Q**). In summary, our study demonstrates that PPFIA1 is highly expressed in pancreatic cancer and promotes malignant phenotypes including proliferation, migration, and invasion. Targeting PPFIA1 may serve as a promising strategy for improving the detection and treatment of pancreatic cancer. EMT core markers and rescue experiments were not performed to validate the mechanistic link between PPFIA1 and EMT.

### Association of PPFIA1 expression with immunotherapy response across cancers

Immunotherapy has emerged as an important therapeutic strategy in multiple malignancies, particularly for advanced-stage tumors. To explore the potential association between PPFIA1 expression and immunotherapy response, we analyzed publicly available datasets from the Tumor Immunotherapy Gene Expression Resource (TIGER). The cohorts used in this study (IMvigor210, VanAllen, and Kim) represent different cancer types, including urothelial carcinoma and melanoma, rather than pancreatic cancer–specific cohorts. The VanAllen and Kim cohorts have small sample sizes, which limits the robustness of the findings.

**Figure 7:**
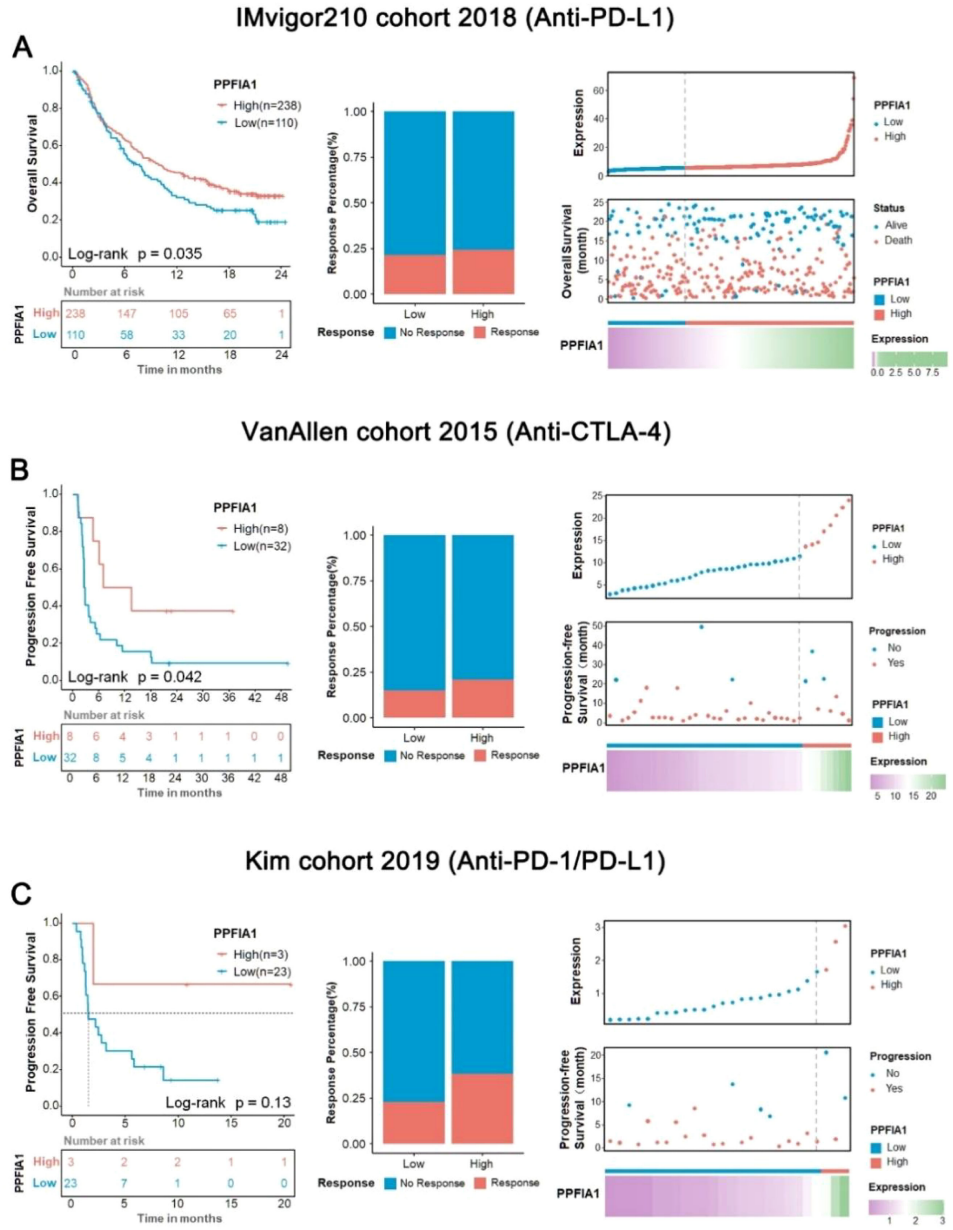
Association of PPFIA1 expression with immunotherapy response across cancers. **(A)** IMvigor210 cohort (anti-PD-L1). **(B)** VanAllen cohort (anti-CTLA-4) **(C)** Kim cohort (anti-PD-1/PD-L1).

As shown in **Figures 7A–C**, patients with higher PPFIA1 expression tended to exhibit improved responses to immune checkpoint blockade therapies across these cohorts. These findings suggest that PPFIA1 expression may be associated with immunotherapy response in a pan-cancer context.

However, given that these datasets are not derived from pancreatic cancer, the interpretation of these results should be approached with caution. The potential role of PPFIA1 as a predictive biomarker for immunotherapy in pancreatic cancer requires further validation in disease-specific cohorts and prospective studies.

## Discussion

In recent years, advancements in surgical techniques for pancreatic cancer, along with the emergence of treatments such as chemotherapy, and radiotherapy have led to encouraging improvements in therapeutic outcomes. However, due to tumor heterogeneity and the lack of reliable biomarkers and therapeutic targets, the overall prognosis of pancreatic cancer still requires significant improvement (29, 30). Therefore, identifying novel biomarkers and targets is of great importance for enhancing the prognosis of PAAD patients.

PPFIA1 is a cytoplasmic scaffolding protein involved in synapse formation and function, playing an important role in neuronal cells (16). Multiple studies have shown that PPFIA1 regulates cell migration and invasion through its influence on pseudopodia (15). Additionally, PPFIA1 participates in cell adhesion, signal transduction, and cytoskeletal organization (12, 31). Studies have indicated that PPFIA1 serves as a prognostic marker associated with poor outcomes in head and neck squamous cell carcinoma and breast cancer (32–34). Furthermore, PPFIA1 is significantly amplified in breast cancer and esophageal squamous cell carcinoma, where it correlates with tumor metastasis and unfavorable prognosis (10, 35, 36). However, there remains a lack of systematic reports on the role of PPFIA1 in pan-cancer analyses or specifically in pancreatic cancer.

With the increasing application of multi-omics technologies in oncology, data mining through multi-omics approaches has become an important means of identifying novel biomarkers (37) (https://doi.org/10.71321/lcflx.00001). This study integrated pan-cancer multi-omics data to comprehensively analyze the role of PPFIA1 across various cancers, including its expression patterns, prognostic significance, genomic alterations, and pathway activities. Further validation was performed in pancreatic cancer to examine PPFIA1 expression, clinical relevance, and functional impact. Overall, PPFIA1 is primarily expressed in cancer cells and exhibits distinct expression patterns across different cancer types. Its high expression is associated with poor prognosis in the majority of malignancies. In most cancer types, PPFIA1 activates the Mitotic Spindle pathway. Notably, PPFIA1 is highly expressed in pancreatic cancer, and only in pancreatic cancer is its high expression consistently associated with poor outcomes across multiple clinical endpoints. Subsequent knockdown of PPFIA1 in pancreatic cancer cell lines impaired cancer cell proliferation, migration and invasion, indicating its potential as both a biomarker and a therapeutic target in pancreatic cancer.

**Figure 8:**
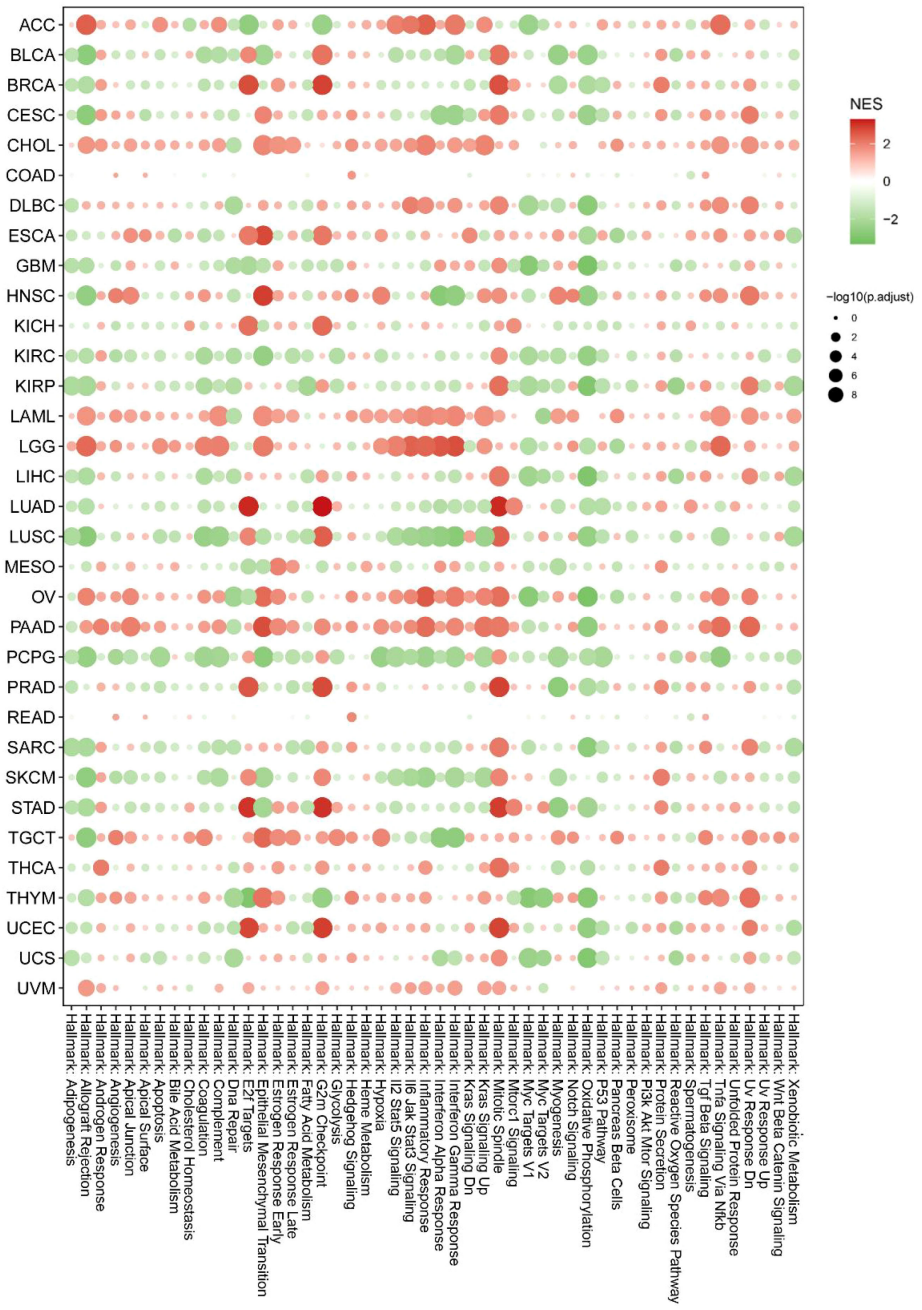
Bubble plot illustrating gene set enrichment analysis (GSEA). Hallmark pathways associated with PPFIA1 expression across pan-cancer cohorts. Cancer types are shown on the y-axis, and hallmark gene sets are represented on the x-axis.

It is noteworthy that we analyzed downstream signaling of PPFIA1 in a pan-cancer context (**Figure 8**). We found that PPFIA1 primarily activates the Mitotic Spindle pathway and suppresses Oxidative Phosphorylation across various tumors. These pathways are frequently altered in cancers and influence tumor progression by regulating cell proliferation and metabolism (38, 39), suggesting a key role for PPFIA1 in cell division and metabolic functions. In pancreatic cancer, the most prominently activated pathway by PPFIA1 was EMT. We further validated the expression and function of PPFIA1 in pancreatic cancer using external datasets, confirming its overexpression and association with poor prognosis. Loss function assays demonstrated that PPFIA1 promotes proliferation, migration, and invasion in pancreatic cancer cell lines. The low survival rate of PAAD can be attributed to its aggressive phenotype, including early local invasion and metastasis (40). Numerous studies have shown that the invasiveness of pancreatic cancer is associated with EMT (41, 42). Therefore, elucidating the molecular mechanisms of EMT is critical for improving patient survival in pancreatic cancer.

Interestingly, we observed that patients with higher PPFIA1 expression showed better responses to immunotherapy in several publicly available cohorts. However, it should be emphasized that these datasets are derived from non-pancreatic cancer types, such as urothelial carcinoma and melanoma. Therefore, the observed association reflects a potential pan-cancer trend rather than a pancreatic cancer–specific phenomenon (43). Given the highly immunosuppressive microenvironment of pancreatic cancer and the limited clinical efficacy of immune checkpoint inhibitors in this disease, the predictive value of PPFIA1 in pancreatic cancer immunotherapy remains uncertain. Further validation in pancreatic cancer–specific cohorts and prospective studies is required.

This study has several limitations. First, although multi-omics analyses were performed across multiple cohorts, independent clinical validation in pancreatic cancer remains lacking. Second, only univariate Cox models were used for survival analysis without adjustment for clinical covariates. Third, the immunotherapy datasets used were not pancreatic cancer–specific and had limited sample sizes, and correlations with TME indicators were not analyzed. Fourth, mechanistic investigations were primarily based on bioinformatic analyses and loss-of-function experiments, without protein-level validation, multiple shRNAs, EMT marker detection, or rescue assays. Fifth, the roles of mitotic spindle and oxidative phosphorylation pathways in pancreatic cancer were not explored. Sixth, the mouse model had a small sample size (n=5 per group). Future studies are warranted to address these limitations.

## Conclusion

Pan-cancer analyses reveal that PPFIA1 is differentially expressed across multiple malignancies and is associated with prognostic outcomes. In pancreatic cancer, PPFIA1 is overexpressed and correlates with poor prognosis, and functional assays suggest its involvement in proliferation, migration, and invasion. Enrichment analyses indicate a potential association with mitotic spindle and EMT-related pathways. Genomic alterations, including amplifications and mutations, are also observed across cancers. Furthermore, PPFIA1 expression may be associated with immunotherapy response in a pan-cancer context. However, given the lack of pancreatic cancer–specific immunotherapy data and independent clinical validation, these findings should be interpreted with caution. Clinical translation and direct application in pancreatic cancer immunotherapy require further validation.

## Data Availability

The raw data supporting the conclusions of this article will be made available by the authors, without undue reservation.
